# Supplementary material for: Immunophenotyping of Peripheral Blood Cells in Patients with Chronic Lymphocytic Leukemia Treated with Ibrutinib
Source: Cells. 2024 Aug 30;13(17):1458. doi: 10.3390/cells13171458 (PMC11393851; doi:10.3390/cells13171458)
Supplement: Supplementary file 1 [file cells-13-01458-s001.zip › cells-3105017-supplementary.pdf]

Table S1. Spectral flow cytometry panel

Reagent	Source	Identifier	Working concentration
Live/Dead Blue	Thermo Fisher Scientific	L23105	1/1000
cFluor® YG584 Anti-Human CD4 (SK3)	Cytek	SKU R7-20041	1/100
Spark NIR™ 685 anti-human CD20 Antibody (2H7)	BioLegend	302366	1/40
APC/Fire™ 750 anti-human CD39 Antibody (A1)	BioLegend	328230	1/80
Pacific Blue™ anti-human CD57 Antibody (HNK-1)	BioLegend	359608	1/80
APC/Fire™ 810 anti-human CD3 Antibody (SK7)	BioLegend	344858	1/80
Vio® Bright FITC anti-human CD120b (TNF-RII) Antibody,REAfinity™ (REA520)	Miltenyi Biotec	130-119-777	1/50
PE/Dazzle™ 594 anti-human TIGIT (VSTM3) Antibody (A15153G)	BioLegend	372716	1/40
PE/Cy5.5 LAG-3 Antibody (17B4)	Novus Biologicals	NBP1-97657PECY55	1/3200
PerCP-Vio® 700 anti-human CD163 Antibody, REAfinity™ (REA812)	Miltenyi Biotec	130-112-133	1/100
PerCP-Cy™5.5 Mouse Anti-Human TCR γδ (B1)	BD Biosciences	564157	1/20
CD137 Antibody, anti-human, APC, REAfinity™ (REA765)	Miltenyi Biotec	130-110-764	1/50
BUV563 Mouse Anti-Human CD7 (M-T701)	BD Biosciences	741355	1/80
Brilliant Violet 570™ anti-human CD45RA Antibody (HI100)	BioLegend	304132	1/40
BV605 Mouse Anti-Human CD56 (NCAM16.2)	BD Biosciences	562780	1/40
BUV805 Mouse Anti-Human CD8 (SK1)	BD Biosciences	612889	1/80
BB515 Mouse Anti-Human CD11c (B-ly6)	BD Biosciences	564490	1/40
BV510 Mouse Anti-Human NKG2A (CD159a) (131411)	BD Biosciences	747922	1/20
BUV661 Mouse Anti-Human CD226 (DX11)	BD Biosciences	749934	1/40
BUV737 Mouse Anti-Human CD134 (ACT35)	BD Biosciences	749286	1/40
BV750 Mouse Anti-Human CD278 (ICOS) (DX29)	BD Biosciences	746858	1/40
BUV615 Mouse Anti-Human TIM-3 (CD366) (7D3)	BD Biosciences	752363	1/20
BV786 Mouse Anti-Human CD279 (PD-1) (EH12.1)	BD Biosciences	563789	1/40
BUV496 Mouse Anti-Human CD10 (MEM-78)	BD Biosciences	750190	1/40
Alexa Fluor® 647 Mouse Anti-TCF-7/TCF-1 (S33-966)	BD Biosciences	566693	1/20
PE-Cyanine5 anti-human FOXP3 Monoclonal Antibody (PCH101)	Thermo Fisher Scientific	15-4776-42	1/40
Alexa Fluor® 700 Mouse anti-Human Granzyme B (GB11)	BD Biosciences	560213	1/80
PE-Cyanine7 anti-human CD152 (CTLA-4) (14D3)	Thermo Fisher Scientific	25-1529-42	1/40
PE Mouse Anti-EOMES (X4-83)	BD Biosciences	566749	1/20
BV650 Mouse Anti-T-bet (O4-46)	BD Biosciences	564142	1/40
BUV395 Mouse Anti-Ki-67 (B56)	BD Biosciences	564071	1/40
